# 913. Impact of BioFire^®^ FilmArray^®^ Blood Culture Identification Panels on Time to Optimal Antimicrobial Therapy

**DOI:** 10.1093/ofid/ofac492.758

**Published:** 2022-12-15

**Authors:** Shivam Vyas, Pamela Giordano, Dimple Patel, Aiman Bandali

**Affiliations:** Atlantic Health System, Morristown, New Jersey; Atlantic Health System, Morristown, New Jersey; Atlantic Health System, Morristown, New Jersey; Atlantic Health System, Morristown, New Jersey

## Abstract

**Background:**

Bloodstream infections are associated with significant morbidity, mortality, and costs. Timely initiation of effective treatment is required for optimal outcomes. Conventional microbiological techniques require 72 hours for organism identification; however, rapid blood culture identification (BCID) polymerase chain reaction (PCR) panels have expedited this process. At Atlantic Health System, the BioFire^®^ FilmArray^®^ BCID panel was implemented June 2020 (BCID1), with subsequent upgrade to BCID2 March 2021. Notable features of the BCID2 panel include speciation of *Enterococcus spp.* and the addition of the CTX-M target. The purpose of this study is to assess time to optimal therapy (TTOT) with and without the BCID platforms for select organisms.

**Table 1:**
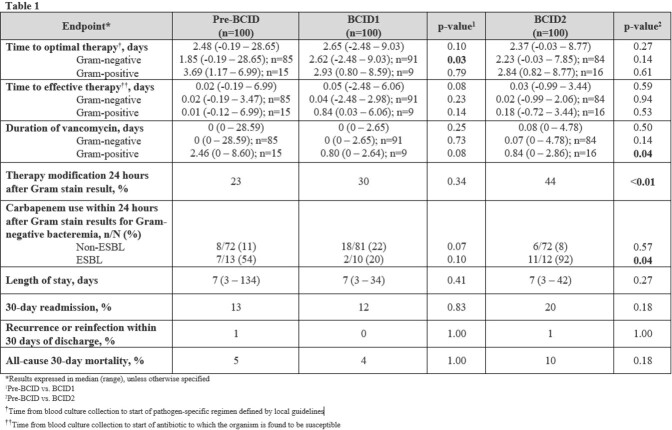
Select Primary and Secondary Endpoints

**Methods:**

This retrospective study of hospitalized, adult patients was conducted at two community teaching hospitals from 5/2019 to 12/2021. Patients with a blood culture positive for *Enterococcus faecalis*, *E. faecium*, *Proteus spp.*, *Escherichia coli*, or non-*aerogenes Klebsiella spp.* were included. Patients with polymicrobial or presumed polymicrobial source of bacteremia, recent positive blood cultures, PCR not performed in BCID arms, or who were deceased/comfort care at time of Gram stain were excluded. The primary endpoint was TTOT. Secondary endpoints included time to effective therapy, duration of vancomycin, therapy modification 24 hours after Gram stain result, and length of stay. All analyses compared PreBCID to either BCID1 or BCID2 arm.

**Results:**

Three hundred patients (100 per arm) were included. Patient characteristics were similar across study arms. The median Pitt Bacteremia Score was 1, the most common organism identified was *E. coli* (65%), the most common source of bacteremia was genitourinary (65%), and 11% of isolates produced ESBL. TTOT (days) was similar in PreBCID compared to BCID1 and BCID2 [(2.48 vs. 2.65, p=0.10); (2.48 vs. 2.37 days, p=0.27)]. TTOT was significantly longer in the gram-negative subgroup of the BCID1 arm compared to PreBCID (2.62 vs 1.85 days, p=0.03). See Table 1 for full analyses.

**Conclusion:**

Implementation of the BCID panels did not reduce TTOT in select organisms at our institution. Barriers to benefit may include limited education and lack of formal antimicrobial stewardship team involvement.

**Disclosures:**

**All Authors**: No reported disclosures.

